# Culturally adapted DBT parent coaching for Chinese families of adolescents with mental disorders: a mixed-methods feasibility study

**DOI:** 10.1186/s40359-026-04932-5

**Published:** 2026-06-10

**Authors:** Anqi Huang, Kaiyan Gan, Jing Xu, YuXin Qian, Jiafang Liu, Jiawei Li, Qian Li, Zenghe Yue, Marcus Rodriguez, Xiaoyan Ke

**Affiliations:** 1 Child Mental Health Research Center, Nanjing Brain Hospital, Clinical Teaching Hospital of Medical School, Nanjing University, 264 GuangZhou Road, Nanjing, 210029 China; 2 Child Mental Health Research Center, The Affiliated Brain Hospital of Nanjing Medical University, Nanjing, 210029 China; 3 Pitzer College, Claremont, CA USA

**Keywords:** Dialectical behaviour therapy, Parent coaching, Adolescent mental health, Emotion regulation, Family functioning, Cultural adaptation

## Abstract

**Background:**

Parents of adolescents with mental disorders frequently experience elevated levels of psychological distress, often in settings where structured support for caregivers is limited. Dialectical Behaviour Therapy (DBT) offers a skills-based framework that may strengthen caregivers’ emotion regulation and improve family functioning, yet culturally adapted parent-focused programmes remain underexamined in China. This study evaluated the feasibility and preliminary effectiveness of a culturally adapted DBT parent coaching programme for Chinese parents of adolescents with mental disorders.

**Methods:**

A mixed-methods, single-arm feasibility design was used, in which 42 parents of adolescents with mental disorders participated in a 10-week culturally adapted DBT parent coaching programme consisting of weekly online group sessions and offline homework practice. Quantitative data were collected at baseline (T1), post-intervention (T2), and three-month follow-up (T3) using validated measures of depression, anxiety, stress (DASS-21), emotion regulation (ERQ), and family functioning (SCORE-15). Paired t-tests and linear mixed-effects models were conducted to examine immediate and longitudinal changes. Qualitative data were obtained through semi-structured interviews with a purposive subsample and analysed using thematic analysis to explore participants’ experiences and perceived changes.

**Results:**

Significant reductions were observed in parents’ depression, anxiety, and stress at post-intervention, alongside improvements in overall family functioning and the strengths and difficulties dimensions of the SCORE-15 (*P* < 0.05). Longitudinal analyses showed patterns consistent with sustained improvement from T1 to T3 in negative emotional symptoms, family functioning, and cognitive reappraisal, with qualitative findings supporting increased use of mindfulness, acceptance, and more constructive communication within families. Interview themes reflected motivations for participation, perceived personal and relational changes, and suggestions for programme refinement.

**Conclusions:**

The findings provide preliminary support for the feasibility and acceptability of a culturally adapted DBT parent coaching programme in the Chinese context. Improvements observed across quantitative and qualitative indicators suggest that the programme may offer a useful, skills-based resource for supporting caregiver wellbeing and aspects of family functioning. Within a cultural environment that places substantial emphasis on parental responsibility yet offers limited formal support, DBT parent coaching appears to be a contextually relevant and potentially valuable approach that warrants further evaluation through controlled and comparative study designs.

**Supplementary Information:**

The online version contains supplementary material available at 10.1186/s40359-026-04932-5.

## Introduction

Parents of adolescents with mental disorders frequently shoulder a substantial and enduring caregiving burden, particularly in settings where professional mental health services are limited in availability, fragmented in organization, or financially inaccessible [1]. Caregivers often experience diminished psychosocial wellbeing, including reduced quality of life and life satisfaction, alongside elevated levels of psychological distress such as depression, anxiety, and stress [2–5]. A recent national survey in China reported that 72.1% of caregivers experienced a high level of burden, with 53.5% reporting moderate to severe depression and 43.1% moderate to severe anxiety [6]. Although international health policy frameworks, including those of the World Health Organization, have increasingly acknowledged the importance of parental mental health during childhood and adolescence, practical implementation has largely focused on parents with diagnosed mental health conditions, rather than recognising parents as recipients of mental health care in their own right [7]. Systematic reviews have demonstrated that family- and parent-focused interventions, particularly those integrating psychoeducation and emotion regulation strategies, can significantly improve adolescent outcomes [8, 9]. However, these approaches remain insufficiently embedded within mainstream mental health systems. These gaps highlight the need for accessible, evidence-informed interventions that support parents’ emotional wellbeing and caregiving capacities within adolescent mental healthcare.

Adolescence is a pivotal stage in the development of emotional, cognitive, and social capacities essential for long-term mental health [10, 11]. Yet globally, mental health conditions during this period have become increasingly prevalent. In China, a national epidemiological survey reported a prevalence of 17.5% among school-aged children and adolescents [12]. Mental disorders emerging during adolescence are frequently associated with long-term impairments, which, if left unaddressed, can result in reduced quality of life, persistent functional limitations, and continued mental health challenges into adulthood [13, 14]. However, child and adolescent mental health services in China remain chronically underdeveloped, with a shortage of trained clinicians and uneven service distribution across regions [15].

Parenting interventions are the cornerstone of adolescent mental health prevention and treatment [16, 17]. Theoretical advances have found that parental emotion socialization, namely parent emotion awareness and regulation, parent reactions to their adolescents’ emotions, and whether parents coach or guide adolescents in recognizing, understanding, and regulating emotions (emotional competence), play a central role in adolescents’ development [18, 19]. The tripartite model of family influences on adolescent emotion regulation further proposes three interrelated processes through which parents influence adolescents’ emotion regulation: (i) observational learning, whereby parents model emotion regulation during social interactions; (ii) specific emotion socialization practices, such as emotion coaching, which shape adolescents’ emotion knowledge and regulatory skills; and (iii) the broader family emotional climate, including parenting behaviours and styles, which influence adolescents’ emotional expression and regulation [16]. Within this framework, the family context, and particularly parental emotion socialization, is considered one of the most influential interpersonal environments shaping adolescents’ emotional development [8, 16, 17]. From a family systems perspective, emotional processes occurring within one family relationship may influence other relationships within the family, highlighting that individual functioning must be understood within the broader family system [20]. Accordingly, parental emotion regulation and family climate are bidirectionally related to adolescent mental health, as adolescents’ psychological difficulties may also contribute to increased parental distress and disruptions in family functioning [1, 21].

A recent systematic review described parents’ lived experiences as “hiding behind walls on fire, engulfed in chaos”, capturing their dual role in crisis management and care navigation while illustrating how easily their needs can be overlooked within routine clinical care [1]. Parents of adolescents with mental disorders may carry self-blame and guilt related to their child’s mental health difficulties, face stigma directed toward both their adolescent and themselves, experience socioeconomic challenges in meeting their adolescent’s needs, feel overwhelmed by limited understanding of their adolescent’s mental illness, and perceive inadequate support from the healthcare system [1]. Parental stress and difficulties in emotion regulation can undermine treatment effectiveness, destabilise family functioning, and perpetuate cycles of emotional conflict [22–24], highlighting the importance of supporting parental wellbeing and emotion regulation capacities for strengthening family resilience and improving adolescent mental health outcomes.

Dialectical behaviour therapy for adolescents (DBT-A), an adaptation of DBT originally developed for young people struggling with severe and pervasive emotional dysregulation, self-harm, and suicidal behaviours, has been demonstrated to reduce self-harm and suicidal ideation and improve emotional functioning in adolescents compared with treatment as usual and other interventions [25–27]. Beyond its effectiveness in adolescents, the theoretical foundations of DBT also support family-focused interventions [28–31]. The theoretical underpinnings of DBT emphasise the importance of relationships between individuals and significant others in their environment, as reflected in the biosocial theory, which proposes that severe and pervasive emotion dysregulation develops and is maintained through ongoing transactions between biological vulnerability to heightened emotional sensitivity and an invalidating social environment [25, 31, 32]. These processes suggest that family interactions may either reinforce or buffer emotional dysregulation and broader mental health difficulties, underscoring the influential role of parental emotion regulation and family dynamics in adolescents’ emotional and psychological development.

Such insights position parents as pivotal agents in therapeutic efforts to transform maladaptive emotional dynamics and promote adolescents’ broader mental health and family functioning [31, 33, 34]. DBT therefore provides a coherent theoretical foundation for family-focused interventions aimed at strengthening caregivers’ emotion regulation capacities, fostering effective communication, and restoring adaptive relational patterns in the home environment. Emerging evidence further suggests that DBT-informed parent interventions may improve parental wellbeing, enhance family functioning, and contribute to better adolescent mental health outcomes [30, 31], with benefits also observed in parent-only DBT interventions [34].

Most psychological interventions, including DBT, have been developed and validated in Western contexts [35]. Evidence suggests that culturally adapted parenting interventions demonstrate improved effectiveness across diverse cultural settings, highlighting the importance of cultural adaptation [36, 37]. Cultural adaptation has been defined as the systematic modification of evidence-based interventions to account for language, culture, and contextual factors, ensuring congruence with the cultural background of service users [35, 38, 39]. DBT is a principle-driven intervention that emphasises flexible application of core therapeutic principles rather than rigid adherence to specific techniques, making it particularly well suited for cultural adaptation across diverse populations. Cultural norms in China differ in important ways [35]. Traditional familism and Confucian ethics place a strong normative emphasis on parental responsibility for adolescents’ development, often intensified by limited formal family support services [6]. In addition, help-seeking from mental health services may be lower among Chinese populations, potentially leading to more severe clinical presentations at treatment initiation and an increased likelihood of poor treatment outcomes or treatment dropout [35]. Furthermore, cultural adaptations increase the efficacy of psychological interventions in adolescents [35, 37], with similar benefits observed in parenting interventions [40], underscoring the importance of culturally adapting DBT-based parent interventions within the Chinese context.

Given these gaps, this study evaluated a culturally adapted DBT parent coaching programme for parents of adolescents with mental disorders in China. Using a mixed-methods design, we assessed changes in parents’ negative emotions, emotion regulation, and family functioning, alongside their subjective experiences of participation. By addressing an often-overlooked dimension of adolescent mental health care, this study aimed to examine the potential value of culturally adapted, skills-based parent interventions for improving parental wellbeing and supporting adolescent mental health in the Chinese context.

## Methods

### Study design

**Fig. 1:**
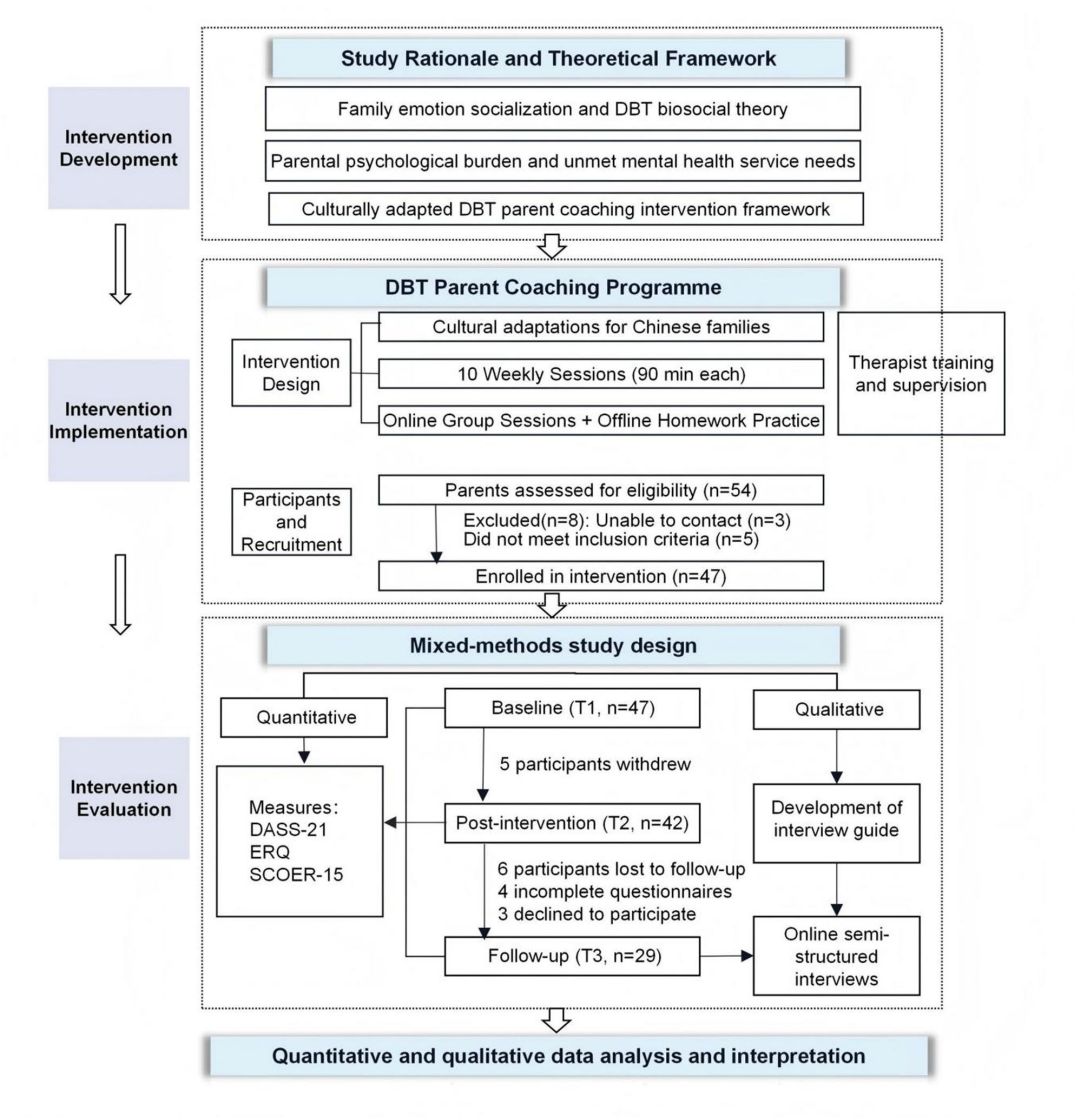
Study design and DBT parent coaching programme procedure

This study employed a mixed-methods, single-arm feasibility design with pre-intervention, post-intervention, and follow-up assessments to evaluate a culturally adapted DBT parent coaching programme for Chinese families of adolescents with mental disorders (Fig. 1). Quantitative analyses assessed changes in psychological distress and family functioning, while qualitative interviews explored parents’ experiences, perceived changes, and suggestions for programme improvement.

### Participants and recruitment

A convenience sampling method was used to recruit parents (either the father or mother) of adolescents with mental disorders who sought treatment at [institution name] between July 2023 and February 2024. Eligible participants were parents of adolescents aged 10 ~ 18 years who had received a formal diagnosis of one or more mental disorders by experienced attending physicians or senior child psychiatrists, according to DSM-5 diagnostic criteria. Parents were required to have sufficient literacy and comprehension abilities to understand intervention materials and participate in group discussions and homework tasks. Participants were also required to be able to use electronic devices, as components of the intervention and assessments were delivered online. These requirements were intended to facilitate engagement with the intervention.

Exclusion criteria included major organic brain diseases, physical disabilities, severe auditory or visual impairments, history of serious head trauma, or substance use disorders. Participants receiving psychotropic medications or psychological therapy were not excluded, provided that treatment remained stable during the intervention period. All participants provided informed consent prior to participation.

A total of 47 parents were enrolled in the DBT parent coaching programme. Complete baseline data were obtained from 42 participants (Week 1), and 29 participants completed post-intervention assessments (Week 12), corresponding to attrition rates of 10.64% and 39.83%, respectively. Attrition was primarily due to scheduling conflicts and competing family responsibilities.

Among the 42 participants included in the quantitative analysis, the mean age of parents was 43.55 ± 5.07 years, and most participants were mothers (30, 71.43%). The majority of families were nuclear families (29, 69.05%), and most parents had a college degree or above (36, 85.71%). The adolescents had a mean age of 13.52 ± 1.99 years, with 18 males (42.86%). Depressive disorders were the most common diagnosis (36, 85.71%), followed by anxiety disorders (23, 54.76%), attention-deficit/hyperactivity disorder (ADHD; 9, 21.43%), and autism spectrum disorder (ASD; 5, 11.90%). A high rate of comorbidity was observed, with 35 adolescents (83.33%) presenting with two or more co-occurring mental disorders.

The qualitative study included 12 participants, of whom 8 (66.67%) were mothers. Given the relatively small number of fathers in the qualitative subsample, gender-based comparisons were not conducted for the qualitative interviews.

### Sample size statement

Sample size estimation was conducted to guide recruitment. Based on previous parenting and DBT-based intervention studies, assuming a medium effect size (d = 0.50), a two-sided significance level of 0.05, and statistical power of 0.80, approximately 34 participants were required to detect pre–post changes. Considering an anticipated attrition rate of 20 ~ 25%, the target recruitment size was set at approximately 45 ~ 50 participants.

### Intervention

The DBT parent coaching programme consisted of 10 weekly group sessions, each lasting approximately 90 min. The intervention was delivered through online group sessions combined with offline homework practice and guidance. Each session followed a structured format to ensure consistency, and the detailed session content is presented in Table 1. The curriculum was adapted from the standard DBT manual [32] and the DBT-A manual [41], with cultural adaptations introduced to enhance relevance and accessibility for Chinese parents while preserving fidelity to DBT’s core modules, including interpersonal effectiveness, mindfulness, emotion regulation, and distress tolerance.

**Table 1:** DBT parent coaching group content

Session	Module	Skills Taught (Online)	Homework Practice (Offline)
Week 1	Introduction	Biosocial theory, programme orientation, self-introduction	Reflect on personal emotional responses and practice validation of children’s emotions
Week 2	Interpersonal Effectiveness	Validation skills	Practice validation skills in parent–child interactions at least three times during the week
Week 3	Interpersonal Effectiveness	DEAR MAN technique	Apply DEAR MAN skills in real-life communication and record experiences
Week 4	Interpersonal Effectiveness	Boundary setting and reinforcement strategies	Complete expectation-setting and behaviour-shaping worksheets
Week 5	Mindfulness	Wise Mind	Identify Wise Mind experiences and reflect on emotional mind, reasonable mind, and Wise Mind
Week 6	Mindfulness	What & How skills	Practice What & How skills and record in mindfulness practice log
Week 7	Emotion Regulation	Functions of emotions	Identify emotions, reflect on their functions, and consider adaptive responses
Week 8	Emotion Regulation	Opposite action	Describe emotions, identify action urges, and practice opposite action
Week 9	Distress Tolerance	STOP, TIP, ACCEPTS	Practice STOP, TIP, and ACCEPTS skills in daily situations
Week 10	Distress Tolerance	Radical acceptance; programme review	Skill consolidation, review, and continued practice planning

### Intervention implementation

Each session followed a standardised structure to enhance consistency across groups. Sessions began with a brief mindfulness exercise and sharing of participants’ experiences (approximately 15 min). This was followed by homework review and guidance (approximately 25 min), during which therapists provided feedback and addressed difficulties encountered in skill application. In the first session, homework review was replaced with participant self-introductions and discussion of personal goals for joining the programme. The core skills teaching component lasted approximately 30 min and focused on DBT-informed parenting strategies. Participants were then divided into six small groups, each consisting of approximately 6–8 parents, to practice skills and discuss real-life parenting scenarios (approximately 20 min). Each small group was facilitated by therapists who were trained psychologists at the Master’s level or above and had received DBT-related training. The session concluded with a summary, reflection, and questions.

Between sessions, participants were assigned homework to encourage application of skills in daily life. Parents were asked to practice skills at least three times per week and submit homework records to therapists. Participants could contact therapists during the week if they encountered difficulties in completing homework, and additional guidance was provided as needed.

To support participant engagement, parents were reminded of each upcoming session one day in advance. Attendance was checked during each session, and parents who had not joined on time were contacted individually by the research team. Homework practice was also followed up weekly, with reminders provided to encourage completion and continued application of skills between sessions. These procedures were used to support intervention implementation, participant engagement, and continuity of skills practice throughout the programme.

### Therapist training and supervision

To ensure intervention fidelity, a standardised intervention manual was developed and used across all sessions. Each module was delivered by the same therapist throughout the intervention to maintain consistency.

Weekly peer supervision meetings were conducted among therapists to review session delivery, discuss participant progress, and address challenges encountered during implementation. In addition, external supervision was provided by an experienced international DBT consultant. The consultant reviewed session plans and provided guidance prior to sessions, particularly regarding parent engagement, skill teaching, and group facilitation strategies.

Therapists also documented session progress, homework submission, and participant engagement using session records. These procedures were used to monitor intervention delivery and ensure consistency across sessions.

### Cultural adaptations

The intervention curriculum was culturally adapted to enhance relevance and acceptability for Chinese parents while maintaining fidelity to core DBT principles. As DBT was originally developed in Western cultural contexts, minor cultural adaptations were considered necessary to improve applicability within Chinese family settings.

The adaptation process involved three steps. First, relevant literature on culturally adapted psychological interventions and DBT parent programmes was reviewed to identify culturally sensitive components. Second, clinicians with experience in DBT delivery and child and adolescent mental health participated in expert discussions to refine the curriculum. Third, participant feedback collected during programme implementation was used to further refine session content and delivery. Specific adaptations included:

- Linguistic adaptation: English metaphors, idioms, and skill examples were replaced with culturally familiar Chinese expressions to improve clarity and resonance.
- Contextualisation of parenting scenarios: Examples were adapted to reflect common Chinese family dynamics, including academic expectations, intergenerational caregiving, and culturally shaped parental responsibility norms.
- Mindfulness integration: Brief mindfulness exercises were embedded into each session to support gradual skill consolidation and accommodate varying levels of prior exposure to mindfulness practices.
- Enhanced focus on validation and Wise Mind skills: These strategies were emphasised during homework and group discussions to align with communication styles and emotional expression patterns commonly observed in Chinese families.

## Research procedure

### Quantitative component

In the quantitative component, self-report questionnaire data were collected at three time points: within one week before the first session (baseline, T1), within one week after the final session (post-intervention, T2), and 12 weeks after programme completion (follow-up, T3).

### Quantitative measures

The Depression Anxiety Stress Scales-21 (DASS-21), developed by Lovibond [42], is a self-report instrument designed to assess the severity of depression, anxiety, and stress. Each of the three subscales consists of seven items rated on a 4-point Likert scale ranging from 0 (not at all) to 3 (very much), reflecting emotional states over the past week. Subscale scores were calculated by summing the corresponding items, and the total score was obtained by summing the three subscale scores, with higher scores indicating greater psychological distress. The Chinese version of the DASS-21 has demonstrated good reliability and validity, with Cronbach’s α coefficients for both the total scale and subscales generally exceeding 0.80 [43, 44]. In the present study, the Cronbach’s α for the total scale was 0.96, with subscale coefficients ranging from 0.86 to 0.90, indicating excellent internal consistency.

The Emotion Regulation Questionnaire (ERQ), developed by Gross and John [45], is a 10-item self-report instrument designed to assess two emotion regulation strategies: cognitive reappraisal and expressive suppression. Items are rated on a 7-point Likert scale ranging from 1 (strongly disagree) to 7 (strongly agree), with higher scores indicating greater use of the corresponding emotion regulation strategy. Subscale scores were calculated by summing the corresponding items. The Chinese version of the ERQ has demonstrated good reliability and validity in previous studies, with Cronbach’s α coefficients of 0.85 for cognitive reappraisal and 0.77 for expressive suppression [46]. In the present study, Cronbach’s α coefficients were 0.89 for cognitive reappraisal and 0.63 for expressive suppression, indicating acceptable internal consistency, particularly given the small number of items in the suppression subscale.

The Systemic Clinical Outcome and Routine Evaluation-15 (SCORE-15) is a standardised self-report instrument used to assess family functioning and evaluate changes following family-based interventions. The scale consists of 15 items across three dimensions: strengths, difficulties, and communication. Responses are rated on a 5-point Likert scale ranging from 1 (strongly disagree) to 5 (strongly agree). Several items are negatively worded and reverse-scored during analysis. Mean scores were calculated for the total scale and each subscale, with higher scores indicating poorer family functioning. The Chinese version of the SCORE-15 has demonstrated acceptable reliability, with a Cronbach’s α of 0.81 for the total scale and 0.60 to 0.73 [47]. In the present study, the total scale demonstrated excellent internal consistency (Cronbach’s α = 0.91), with subscale reliability coefficients ranging from 0.69 to 0.88.

### Qualitative component

For the qualitative component, participants were recruited using a maximum variation purposive sampling strategy informed by quantitative outcomes. Parents who had provided consent for follow-up interviews were contacted after completing the programme. To ensure confidentiality, identification numbers were used in place of names.

Telephone interviews were conducted by a researcher who was familiar with DBT but was not involved in delivering the intervention. Each interview lasted approximately 20–30 min and was audio-recorded and transcribed verbatim. Interviews followed a semi-structured guide but were flexibly administered to accommodate the natural flow of participants’ narratives. The interviews explored parents’ experiences of the programme, perceived changes in emotional or relational patterns, and suggestions for improving programme delivery.

Recruitment continued until data saturation was reached, defined as the point at which no new themes or substantial new information emerged [48].

### Interview guide

Semi-structured interviews were conducted to explore parents’ experiences and feedback regarding participation in the DBT parent coaching programme. The interview guide was initially developed based on study objectives, relevant literature on parent interventions, and the clinical experience of the research team, and was subsequently refined through iterative team discussions. Pilot interviews were conducted to assess clarity, logical flow, and relevance of the questions, and minor revisions were subsequently made based on feedback, including refinement of wording and addition of probing questions. The final semi-structured interview guide ensured consistency across interviews while allowing flexibility for in-depth exploration of participants’ experiences. The interview guide is provided in Supplementary File 1.

### Data analysis

#### Quantitative analysis

Statistical analyses were conducted using SPSS 26.0. Continuous variables were presented as mean ± standard deviation, while categorical variables were summarized using frequencies and percentages. Paired-sample t-tests (T1 vs. T2) were conducted to assess immediate post-intervention effects.

Missing data due to participant drop-out were handled using available-case analysis. Only participants who completed the relevant assessments were included in the analyses. To examine longitudinal changes across T1, T2, and T3, a linear mixed-effects model (LMM) was applied. LMMs were selected over repeated-measures ANOVA due to their ability to accommodate missing data and model individual variation over time. Time was specified as a fixed effect, and participant-specific random intercepts were included to account for baseline heterogeneity. *P* < 0.05 was considered statistically significant.

#### Qualitative analysis

Interview recordings were transcribed verbatim within 24 h. Data were analysed using Colaizzi’s seven-step method in NVivo 14. The first and second authors independently coded the transcripts, extracted significant statements, formulated meanings, and clustered themes and subthemes. Discrepancies were discussed with the corresponding author and the third author until consensus was reached. The research team held iterative discussions to review and refine themes, focusing on the semantic features of the data. Throughout the data collection and analysis phases, the seventh author engaged in reflexive practice, including maintaining a reflective journal and examining potential biases. Finally, themes were reviewed against the original transcripts to ensure credibility in accordance with Colaizzi’s method [49].

## Results

### Quantitative results

#### Comparison of differences before and after the intervention

Following the intervention, the results of paired-sample t-tests (Table 2) indicated that the total DASS-21 score, as well as scores for its three subscales (depression, anxiety, and stress), were significantly lower than baseline levels (*t*=-2.24~-2.94, *P* < 0.05). Similarly, the total SCORE-15 score and its strengths and difficulties dimensions showed significant reductions following the DBT parent coaching programme(*t*=-2.20~-2.98, *P* < 0.05), indicating improved family functioning. However, no statistically significant differences were observed in the SCORE-15 communication dimension, the ERQ total score, or its subscales.

**Table 2:** Changes in outcome measures from baseline to post-intervention

Measure	Pre- And Post-Intervention Comparison (M ± SD)		t-value	*P*-value
	T1(*n* = 42)	T2(*n* = 42)		
DASS-21 Total Score	15.02 ± 12.37	9.69 ± 6.93	-2.64	0.012
DASS-21 Depression	4.64 ± 4.02	2.86 ± 2.53	-2.47	0.018
DASS-21 Anxiety	4.21 ± 4.06	2.43 ± 2.06	-2.94	0.005
DASS-21 Stress	6.17 ± 4.94	4.40 ± 3.29	-2.24	0.031
ERQ Total Score	47.57 ± 8.20	50.26 ± 7.85	1.70	0.097
ERQ Cognitive Reappraisal	29.33 ± 7.30	31.43 ± 6.22	1.88	0.067
ERQ Expressive Suppression	18.24 ± 4.34	18.83 ± 4.43	0.71	0.482
SCORE-15 Total Score	2.57 ± 0.70	2.31 ± 0.66	-2.48	0.017
SCORE-15 Strengths	2.48 ± 0.68	2.27 ± 0.70	-2.20	0.033
SCORE-15 Difficulties	2.70 ± 0.92	2.33 ± 0.76	-2.98	0.005
SCORE-15 Communication	2.52 ± 0.74	2.34 ± 0.72	-1.43	0.161

### Longitudinal effects of the DBT parent coaching intervention

The linear mixed-effects model incorporating follow-up data revealed a sustained reduction in the DASS-21 total score and its subscales from T1 to T3, indicating lasting improvements in emotional symptoms(*P* < 0.05). For the ERQ, while the total score increased from T1 to T2, this change was not statistically significant. However, a significant increase was observed from T2 to T3(*P* < 0.05). Notably, the cognitive reappraisal subscale exhibited a consistent upward trend from T1 to T3 (*P* < 0.05), suggesting a progressive improvement in adaptive emotion regulation strategies over the course of the intervention. Regarding SCORE-15, the total score and the strengths and difficulties dimensions showed significant reductions over time(*P* < 0.05), indicating improved family functioning. However, no statistically significant change was detected in the communication dimension.The full results of the linear mixed-effects model are presented in Table 3.

**Table 3:** Linear mixed-effects model results

Measure	T1 Intercept	T2 Change(95%CI)	T3 Change(95%CI)
DASS-21 Total Score	14.19	-4.59 [-8.04, -1.14]**	-7.9 [-11.71, -4.09]***
DASS-21 Depression	4.38	-1.53 [-2.8, -0.19]*	-2.76 [-4.04, -1.49]***
DASS-21 Anxiety	3.96	-1.58 [-2.58, -0.58]**	-2.49 [-3.68, -1.30]***
DASS-21 Stress	5.85	-1.51 [-2.89, -0.13]*	-2.61 [-4.16, -1.06]**
ERQ Total Score	47.72	2.56 [1.09, 4.04]	3.70 [2.13, 5.27]*
ERQ Cognitive Reappraisal	29.30	2.12 [1.04, 3.19]*	3.08 [1.90, 4.25]*
ERQ Expressive Suppression	18.43	0.45 [-0.14, 1.04]	0.71 [0.08, 1.33]
SCORE-15 Total Score	2.56	-0.25 [-0.49, -0.02]**	-0.30 [-0.55, -0.04]**
SCORE-15 Strengths	2.49	-0.22 [-0.43, -0.00]*	-0.42 [-0.66, -0.17]***
SCORE-15 Difficulties	2.67	-0.35 [-0.53, -0.18]**	-0.36 [-0.56, -0.15]**
SCORE-15 Communication	2.53	-0.18 [-0.45, 0.08]	-0.13 [-0.41, 0.15]

T1 = Baseline, T2 = Post-Intervention, T3 = Follow-Up

*P < 0.05, **P < 0.01, ***P < 0.001

### Exploratory comparisons between fathers and mothers

Prior to examining intervention effects, exploratory analyses were conducted to assess potential differences between fathers and mothers. No significant differences were observed in DASS-21 scores, SCORE-15 scores, or ERQ scores, including the expressive suppression subscale, at baseline or post-intervention (all *P* > 0.05). A significant difference was observed only in post-intervention ERQ cognitive reappraisal, with mothers scoring higher than fathers (Z=-1.98, *P* = 0.049).

### Qualitative analysis results

**Fig. 2:**
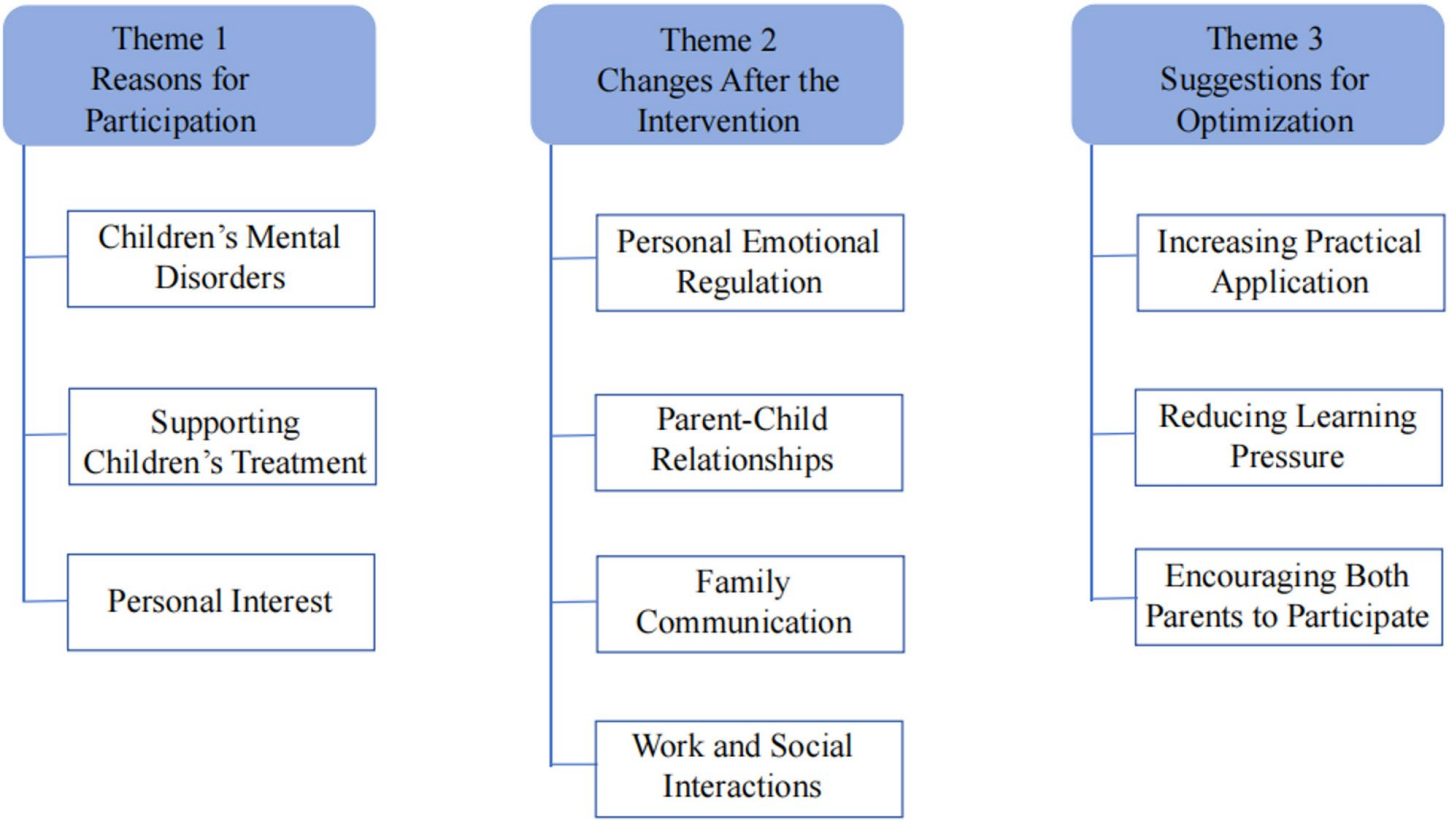
Thematic map of the themes and subthemes

A total of 12 parents participated in the interviews, coded as P1-P12. The analysis of the semi-structured interviews identified three main themes and their corresponding subthemes, as presented in Fig. 2.

### Theme 1. Reasons for participation

#### Children’s emotional difficulties 

All parents reported that their children were experiencing emotional and behavioral difficulties, expressing hope that the course would be helpful in addressing these challenges: *“My child has depression and is currently on leave from school. I hope to apply the knowledge learned in this course to help them.”*(P1);*“My child has frequent emotional outbursts and struggles with emotional regulation*,* along with some social difficulties.”*(P5).

#### Supporting children’s treatment

Many parents enrolled in the DBT parent coaching programme alongside their child’s ongoing treatment, aiming to enhance therapeutic outcomes and provide additional support:*“I want my child’s treatment to be more effective.”*(P6).

#### Personal interest

Some parents expressed an intrinsic interest in the DBT parent coaching programme and were motivated to participate out of curiosity.*“I saw your recruitment notice and found it quite interesting*,* so I decided to enroll.”*(P6).

### Theme 2. Changes after the intervention

#### Parental emotion regulation 

The most immediate change reported by parents was an improvement in their own emotional regulation. Mindfulness and acceptance were the most frequently mentioned skills, helping parents develop greater self-awareness and emotional resilience.*“Mindfulness is particularly valuable. I think the greatest impact of DBT is helping me objectively recognize my emotional state*,* especially when facing difficulties*,* and understand how to perceive myself correctly.”*(P3);*“I feel that the course benefited me the most by teaching me how to communicate and find better ways to regulate myself.”*(P10).

#### Family communication 

Parents reported that shifts in their perspectives led them to adopt new communication strategies, improving overall family relationships. Enhanced emotional recognition enabled them to engage in more constructive interactions with family members:*“It helped me accurately recognize my emotions*,* identify them*,* and then apply what I learned in class. I also tried to communicate better with my husband.”*(P6);*“It’s not just about improving communication with my child*,* it also helps in managing my relationship with my spouse.”*(P9).

#### Parent-child relationships 

Changes in parental thinking and emotional regulation directly influenced parent-child communication, leading to potential improvements in adolescents’ emotional management. Parents learned to respond to conflicts more thoughtfully, reducing reactive emotional outbursts:*“By regulating my own emotions*,* I stopped lashing out at my child*,* which prevented them from having emotional outbursts in response.”*(P9).

#### Work and social interactions

The skills acquired from the DBT parent coaching programme also helped parents manage work-related stress and improve interpersonal relationships beyond the family setting.*“In such a highly competitive work environment… no one has a perfectly smooth career. We all encounter setbacks*,* and I’ve learned to accept the reality of my work situation.”*(P8).

### Theme 3. Suggestions for optimization

Participants recognized the value of the therapists and course structure, expressing appreciation for the interactive and supportive learning environment.

*“The instructors were excellent*,* guiding us through practice and explaining techniques in depth. The group leader was particularly proactive and engaging*,* allowing us to share our thoughts and emotions.”*(P10).

At the same time, parents suggested areas for improvement, including enhanced practical application, reduced stress during classes, and joint parental participation.

#### Increasing practical application 

Parents highlighted the importance of integrating DBT skills into daily life and reinforcing learning through consistent practice. They also recommended incorporating case-based discussions to provide practical guidance tailored to common parenting challenges:*“After class*,* we need to reflect*,* repeatedly apply the skills*,* and develop a habitual way of thinking. Daily practice is essential”*(P12);*“It would be helpful to discuss common challenges faced by most children and explore specific strategies for parents to address these issues.”*(P2).

#### Reducing learning pressure 

Parents recommended fostering a more relaxed, pressure-free learning environment to enhance engagement and support sustained learning outcomes:*“There should be no pressure in class*,* no mental burden… Sometimes*,* what seems ‘useless’ at first may turn out to be incredibly valuable”*(P4); *“Do not hasten results; trust that transformation is a gradual and continuous process*,* but remain committed to completing each lesson.”*(P6).

#### Encouraging both parents to participate 

Parents highlighted the importance of shared learning between both caregivers, noting that mutual growth can foster deeper understanding and cohesion within the family:*“Ideally*,* both parents should take this course together. However*,* if that’s not possible*,* the participating parent should avoid criticizing or blaming the one who didn’t attend.”*(P12).

## Discussion

This study demonstrated that DBT parent coaching was associated with measurable improvements in three key areas: reductions in negative emotional states such as depression, anxiety, and stress; enhanced emotion regulation capacities; and strengthened family functioning. These findings are consistent with previous research on DBT-based parent interventions and further support their value in promoting caregiver wellbeing and improving relational dynamics within families [31, 33]. These quantitative improvements were further supported by qualitative findings, in which parents reported increased emotional awareness, improved family communication, more adaptive parent-child interactions, and the application of DBT skills in broader work and social contexts. As the first evaluation of such a programme in mainland China, incorporating a mixed-methods design and follow-up assessment, this study provides preliminary evidence for the feasibility and contextual relevance of DBT parent coaching in supporting caregivers within adolescent mental health care.

Consistent with prior evidence, this study found that participation in DBT parent coaching programme was associated with marked reductions in parents’ levels of depression, anxiety, and stress. These findings are noteworthy given meta-analytic evidence showing that parents of adolescents with mental disorders often experience substantially higher psychological burden than parents of typically developing youth [50]. Rather than focusing on disorder-specific symptoms, DBT emphasises the transdiagnostic process of emotion dysregulation, which is recognised as a central contributor to a wide range of psychological difficulties [51–53]. This transdiagnostic focus may be particularly relevant for parents caring for adolescents with diverse mental health conditions, as observed in the present study, where adolescents presented with heterogeneous diagnoses and high levels of comorbidity. By providing parents with skills such as mindfulness, distress tolerance, and radical acceptance, the intervention may strengthen emotional flexibility and increase confidence in managing challenging caregiving situations. This interpretation is consistent with emotion regulation theory, which highlights the roles of heightened emotional awareness and cognitive reframing in reducing emotional reactivity and promoting psychological wellbeing [54]. Moreover, this perspective aligns with the biosocial model underlying DBT [25, 31, 32], which proposes that improved emotion regulation skills can reduce reactive responses and foster more adaptive interpersonal interactions. Through these mechanisms, DBT parent coaching may help parents move from reactive patterns of distress toward more regulated and adaptive caregiving behaviours, which may in turn contribute to a more stable and supportive family environment. Such changes in caregiver responses may be particularly important in families of adolescents with emotional and behavioural difficulties, where parental emotional reactions can influence family dynamics and adolescents’ adjustment.

Although the pre–post comparisons did not show statistically significant changes in the use of emotion regulation strategies, the linear mixed-effects model revealed a gradual increase in DBT-related skill use over time. These findings were further supported by qualitative accounts, with many parents describing the incorporation of mindfulness and acceptance strategies into their daily lives. These observations are consistent with previous research suggesting that the acquisition and generalisation of DBT skills contribute meaningfully to therapeutic progress [33, 55]. This pattern suggests that improvements in emotion regulation may emerge gradually rather than immediately following the intervention. Such delayed effects are consistent with the nature of DBT skills, which typically require sustained practice and gradual integration into daily life before measurable behavioural changes become evident. Previous studies have similarly reported that increases in DBT skill use may occur progressively and are associated with later improvements in emotional functioning [31]. Mindfulness practice, in particular, is understood to promote nonjudgmental awareness and emotional attunement, processes that can strengthen regulatory capacity through more effective cognitive reappraisal [56]. Over time, these skills may enable parents to pause before reacting, respond more flexibly to emotionally challenging situations, and reduce reactive patterns of caregiving. Neurobiological models offer further support by proposing that such regulatory processes may facilitate greater top-down modulation of amygdala activity via engagement of prefrontal regions, which provides a plausible explanatory framework for the improvements in emotional functioning observed among caregivers [57, 58]. Taken together, these findings suggest that DBT parent coaching may support progressive improvements in emotion regulation, which may subsequently contribute to broader changes in family functioning and relational dynamics.

Beyond individual outcomes, the intervention was associated with meaningful improvements in overall family functioning at both post-intervention and follow-up assessments. Quantitative results showed increases in family strengths and reductions in relational difficulties, while qualitative accounts described shifts in parenting beliefs, more constructive communication, and greater emotional attunement within the family, as well as improvements in parent-child interactions and reductions in emotionally reactive responses, suggesting that changes in parental emotion regulation may have influenced broader family dynamics. These patterns are consistent with the biosocial model, which emphasises how emotionally invalidating environments can contribute to emotional dysregulation, and they suggest that DBT parent coaching may support the development of more validating and responsive family interactions [28–31]. By enhancing caregivers’ emotion regulation and communication skills, the intervention may contribute to a more supportive and emotionally responsive family environment. Such changes may be particularly important for adolescents experiencing emotional and behavioural difficulties, as family interactions play a central role in shaping adolescents’ emotional regulation and psychological adjustment [16, 17]. Improved parental emotion regulation and family communication may therefore indirectly support adolescents’ treatment engagement, emotional stability, and overall functioning.

This interpretation is also consistent with the tripartite model of family influences on adolescent emotion regulation, which proposes that parents influence adolescents’ emotional development through modelling, emotion coaching, and the broader emotional climate of the family [16]. Improvements in parental emotion regulation may have enhanced modelling processes, whereby adolescents learn adaptive emotional responses through observing their caregivers. Parents described becoming more mindful, less reactive, and more emotionally aware, suggesting that the intervention may have provided adolescents with more stable emotional models. These changes may also reflect improvements in emotion coaching and skill transmission. Parents reported learning validation strategies and more constructive communication, which may help shape adolescents’ emotion knowledge and regulatory skills and promote more adaptive coping responses. Several parents also described improvements that extended beyond the parent–child relationship, including more harmonious marital interactions and smoother everyday family communication. These findings suggest broader improvements in the family emotional climate, highlighting the wider systemic potential of caregiver-focused interventions.

In addition, the present findings also support the notion that cultural adaptations may provide additive benefits to evidence-based interventions by aligning the conceptualisation, structure, and delivery of psychological programmes with culturally relevant values and practices [35]. Such adaptations are consistent with established frameworks for culturally adapting psychological interventions, which emphasise the importance of modifying language, contextual examples, and delivery approaches to enhance cultural relevance and acceptability. The use of culturally adapted examples and group-based discussions in the present study may have further enhanced the acceptability and feasibility of the intervention. Parents described applying skills in everyday family situations, suggesting that DBT-based strategies were transferable to real-life interactions. These findings provide preliminary evidence supporting the feasibility and contextual relevance of culturally adapted DBT parent coaching in mainland China.

These findings further highlight the potential value of culturally adapted, family-focused interventions in settings where formal support for caregivers remains limited. The observed improvements in parental mental health, emotion regulation, and family functioning suggest that DBT parent coaching may offer a useful approach for addressing caregivers’ emotional needs within the broader context of adolescent mental health care. In contexts where parents often assume substantial caregiving responsibility yet structured support remains limited, such programmes may represent a feasible and contextually appropriate means of strengthening caregiver wellbeing. The findings also point to the potential benefits of incorporating family-oriented interventions into community- and school-based mental health services, which may help enhance the continuity and accessibility of support available to adolescents and their families.

### Strengths and limitations

This study provides initial empirical support for the feasibility and potential effectiveness of DBT parent coaching in a Chinese context, demonstrating improvements in parental wellbeing, emotion regulation, and family functioning. By integrating quantitative and qualitative data, the study offers a more comprehensive understanding of parents’ experiences and perceived changes. It also contributes to the growing literature on culturally adapted, skills-based interventions and highlights their potential value in strengthening family processes within adolescent mental health care.

Several limitations should be considered when interpreting these findings. First, the absence of a control group limits the ability to draw causal conclusions regarding the observed changes. Without a comparison group, it is difficult to rule out alternative explanations, such as natural changes over time, regression to the mean, or external influences. Future studies incorporating controlled or randomized designs are therefore needed to allow for more rigorous evaluation of intervention effectiveness. Second, the study did not include adolescents’ perspectives regarding changes within the family, which may have provided a more comprehensive understanding of family-level outcomes and intergenerational dynamics. Future research incorporating multi-informant data, including adolescents and clinicians, may help further clarify the broader impact of parent-focused interventions. Limitations related to participant recruitment should also be acknowledged. Parents were required to have sufficient literacy, comprehension abilities, and access to electronic devices to participate in the intervention, resulting in a relatively highly educated sample. This may limit the generalizability of the findings to families with lower educational attainment or fewer resources, who may face additional barriers to engagement and may require different forms of support. Furthermore, adolescents in this study presented with a range of mental disorders, with a high rate of comorbidity. Due to the relatively small sample size and clinical heterogeneity, the study did not examine whether intervention effects varied according to diagnostic type or disorder severity. Future research with larger and more diverse samples is needed to explore whether intervention outcomes differ across diagnostic profiles and levels of clinical severity.

## Conclusion

This study provides preliminary evidence that culturally adapted DBT parent coaching is a feasible and potentially effective approach for reducing caregiver distress, enhancing emotion regulation, and improving family functioning within the context of adolescent mental health care. Improvements observed across quantitative and qualitative indicators suggest that structured, skills-based support for parents may contribute to meaningful changes at both the individual and family levels.

In settings where parental responsibility is substantial and formal support remains limited, such interventions may offer an accessible and contextually appropriate resource for strengthening caregiver wellbeing. By equipping parents with practical strategies to manage stress, regulate emotions, communicate more effectively, and support healthier family interactions, the programme may help foster relational environments that are more conducive to adolescent recovery and adjustment. As a scalable and flexible approach, culturally adapted DBT parent coaching also shows potential for integration into community- and school-based mental health services, where it may enhance the accessibility and continuity of support available to adolescents and their families.

## Supplementary information

Supplementary Material 1.

## Data Availability

The datasets used and/or analysed during the current study are available from the corresponding author on reasonable request.
