# Supplementary material for: Culturally adapted DBT parent coaching for Chinese families of adolescents with mental disorders: a mixed-methods feasibility study
Source: BMC Psychol. 2026 Jun 10;14:1193. doi: 10.1186/s40359-026-04932-5 (PMC13479578; doi:10.1186/s40359-026-04932-5)
Supplement: Supplementary file 1 [file 40359_2026_4932_MOESM1_ESM.docx]

**Supplementary File 1. Semi-structured Interview Guide**

**Interview introduction**

Thank you very much for participating in this interview. We are researchers from Child Mental Health Research Center, Nanjing Brain Hospital. This interview aims to gather your feedback on the DBT Parent Coaching Programme to help improve the programme and better support parents and their children.

All information collected during the interview will be kept confidential and anonymised for research purposes only. The interview is expected to last approximately 20 minutes.

With your permission, the interview will be audio-recorded to ensure accuracy. You may request a break at any time, and you are free to withdraw from the interview at any point without providing a reason.

**Interview questions**

**1.Motivation for participation** What initially motivated you to participate in the DBT Parent Coaching Programme? Have these concerns or difficulties changed after the programme? If yes, in what ways?

**2.Skill acquisition and application**  Which skills from the programme do you remember learning? Which skills have you used most frequently? In what situations did you use these skills?

(Prompts: validation, mindfulness, distress tolerance, emotion regulation, interpersonal effectiveness)

**3.Perceived benefits**  Based on your overall experience, what benefits did the DBT Parent Coaching Programme bring to you and your child?

**4.Challenges during participation**  Did you encounter any difficulties while participating in the programme? If yes, what were they?

**5.Helpful components**  Which aspects of the programme did you find particularly helpful? Why?

**6.Suggestions for other parents**  What advice would you give to another parent who is considering joining this programme?

**7.Suggestions for programme improvement**  Do you have any suggestions for improving the programme or for the therapists delivering the intervention?

That concludes our interview questions. We would like to extend our sincere thanks for your time, support, and cooperation throughout this interview. Before we finish, is there anything else you would like to share that we have not yet discussed? If any part of today’s conversation has caused discomfort or distress, please feel free to contact us. If needed, we can also provide information about appropriate support services.
